# Cyclooxygeanse-2 promotes metastasis in osteosarcoma

**DOI:** 10.1186/s12935-015-0220-2

**Published:** 2015-07-04

**Authors:** Liyan Qu, Bing Liu

**Affiliations:** Clinical Laboratory Centre, 2nd Affiliated Hospital, School of Medicine, Zhejiang University, #88 Jie Fang Road, Hangzhou, 310009 Zhejiang People’s Republic of China; Clinical Laboratory Centre, Binjiang Hospital of Hangzhou, Hangzhou, Zhejiang China; Department of Orthopedics, 2nd Affiliated Hospital, School of Medicine, Zhejiang University, #88 Jie Fang Road, Hangzhou, 310009 Zhejiang People’s Republic of China

**Keywords:** COX-2, COX-inhibitors, Metastasis, Osteosarcoma

## Abstract

Cyclooxygenase-2 (COX-2), an inducible form of the enzyme that catalyzes the first step in the synthesis of prostanoids, is associated with carcinogenesis, which is suspected to promote angiogenesis and tissue invasion of tumors and resistance to apoptosis. COX-2 is also involved in metastasis and poor prognosis of cancer. Osteosarcoma with COX-2 positivity is from 67 to 92 %. COX-2-positive rate in metastatic lesions was greater than that of biopsy and/or resected samples of the primary site in osteosarcoma. And, what role does COX-2 play in osteosarcoma metastasis? Genetic studies support a cause-effect connection between COX-2 and tumorigenesis. COX-2 expression had a poor prognosis with regard to metastasis, and patients with increased COX-2 expression in lung metastases died of the disease. COX-2 expression has also been established as a marker in human osteosarcoma, and COX-2 inhibition has been suggested as a possible way of improving therapeutic outcome. In addition, COX-inhibitors inhibit the tumor initiation, matrix metalloproteinases (MMPs), cell differentiation and T cell proliferation and suppression of the antitumor activity of natural killer cells and macrophages, angiogenic mechanism. Therefore, we can exert the COX-inhibitors to potentialize the effects of chemotherapeutic agents, and reverse the metastasis in osteosarcoma to facilitate the patient who may benefit from addition of COX-inhibitors to standard cytotoxic therapy.

## Introduction

Cyclooxygeanse-2 (COX-2) is overexpressed in most solid tumors, such as colorectal, liver, pancreatic, breast, lung cancer as well as osteosarcoma [1–6]. The activity of COX-2 is suspected to promote angiogenesis, tissue invasion of tumors [7], metastasis [8, 9], and resistance to apoptosis [10, 11]. Genetic studies support a cause-effect connection between COX-2 and tumorigenesis. Therefore, we can exert the drugs to affect COX-2 and achieve the therapies of human malignancies. Both non-selective non-steroidal anti-inflammatory drugs (NSAIDs), and selective COX-2 inhibitors can inhibit proliferation, invasiveness of tumors.

Osteosarcoma is the most common primary bone tumor generally affecting children and young adults which has been reported to express COX-2 constitutively. Approximately 20 % of patients present with lung metastases at initial diagnosis, additionally, in 40 % of patients metastases occur at a later stage. As we know, osteosarcoma with COX-2 positivity is from 67 to 92 % [9, 12, 13]. Dickens et al. [12] reported the COX-2-positive rate in metastatic lesions was greater than that of biopsy and/or resected samples of the primary site in osteosarcoma. And, what role does COX-2 play in osteosarcoma metastasis?

## Cyclooxygenase

The cyclooxygenases (COX) are enzymes, known as prostaglandin (PG) rate-limiting synthase, catalyze the metabolism of arachidonic acid (AA) to PGs. Finally, a series of biologically active prostaglandins (PGD2, PGE2, PGF2α, and PGI2) and thromboxane A2 (TXA2) are formed. There are three isoforms of the enzyme that have been identified: COX-1, COX-2, and COX-3 [14]. COX-1 is considered a “housekeeping enzyme”, constitutively expressed in human cells. COX-3, an alternate splice variant of COX-1, is most abundant in the canine cerebral cortex. COX-2 is an inducible enzyme and is associated with inflammatory diseases and carcinogenesis, which is suspected to promote angiogenesis and tissue invasion of tumors [7, 15].

## Molecular factors in metastatic osteosarcoma

The metastatic cancer cells subsequently complete the following steps: Invasion through the extracellular host matrix and entrance into the circulation (I), survival in the circulation (II) and evasion of the host immune system (III), arrest and extravasation at a target organ site (IV), adherence and survival in the target organ microenvironment (V, VI) and finally formation of neovasculature to allow growth at the target organ site (VII) [16–21]. PosthumaDeBoer J [16] reported that there are many molecular alterations as target for therapy in metastatic osteosarcoma: (I) Migration and invasion MMPs, m-Calpain, Wnt, Src, Notch; (II) a Anoikis resistance PI3K/Akt, Src/PI3k/Akt, Src/Ras/MAPK, NF-κB, Wnt/β-catenin, BcL family, (II) b Apoptosis resistance Src, NF-κB, Wnt/β-catenin, Fas/FasL; (III) Evasion of immune system HLA-1, IL-10, Fas; (IV) Arrest and extravasationCXCR4-CXCL12,CXCR3-CXCL9-11, CXCR4/MMPs, CXCR3-4/Erk/NF-κB; (V) Adherence Ezrin/MAPK/Akt, Ezrin/β4-Integrin/PI3K, CD44/Akt/mTOR, (VI) Dormancy Integrin-α5β1, Integrin-α5β1/Erk/p38, Bcl-XL, IGF/PI3K, ECM; (VII) Angiogenesis and proliferationEGFR. PDGFR, VEGF, IGFR, TGF-β, MMPs, VEGF/Erk/NF-κB, VEGF/PI3K, EGFR/Src/Ras/MAPK/STAT3, Src, Integrin/PI3K/Erk1-2, Wnt/β-catenin/CyclinD-Survivin.

## COX-2 promotes metastasis in osteosarcoma

COX-2 overexpression in osteosarcoma increases cell mobility and invasiveness, which correlates with the occurrence of distant metastasis in patients with osteosarcoma and also may affect post-metastatic survival [8].

The cancer stem cells (CSCs) share several characteristics with embryonic and somatic stem cells including self-renewal and differentiation abilities, and represent a small fraction of the cellular population of the tumor. Osteosarcoma CSCs have been identified in humans and dogs suggesting that these cells may be responsible for treatment failure in this disease [22, 23]. Pang LY [24] reported that global transcriptional analysis and comparison with parental cells identified COX-2 expression to be significantly increased in this population. They identified that COX-2 was expressed 141-fold more in CSC spheres than daughter adherent cells. Meanwhile, COX-2 expression is elevated in cancer stem cells, which is required for tumoursphere formation, and tumourspheres increased invasiveness and tumourigenicity. They found that COX-2 inhibition had no effect on CSC growth, or resistance to chemotherapy. However inhibition of COX-2 in daughter cells prevented sphere formation, indicating a potential significant role for COX-2 in tumor initiation.

Invasion is mediated mainly by matrix metalloproteinases (MMPs). Among them, MMP-2 (gelatinase A) and MMP-9 (gelatinase B) are known to be strongly correlated with metastatic potential of cancer cells [25]. Lee EJ [26] reported that COX-2 overexpression enhanced mobility and invasiveness of U2OS cells, which was accompanied by increases of matrix metalloproteinase-2 and -9 (MMP-2 and -9) activities. Selective COX-2 inhibitors, NS-398 and celecoxib, inhibited cell proliferation and abrogated the enhanced mobility, invasiveness and MMP activities induced by COX-2 overexpression.

In our study, we reported that Celecoxib, a cyclooxygenase-2 inhibitor, induces apoptosis in human osteosarcoma cell line MG-63 via down-regulation of PI3K/Akt [27]. PI3K/Akt plays an essential role in the cell/extracellar matrix (ECM) and cell/cell adhesion. Lack of the correct adhesion, the adhesion-dependent signals will be interrupted, which will result in adhesion-related apoptosis: anoikis [28]. Activation of PI3K/Akt signaling has been shown to mediate survival signals triggered by the engagement of E-cadherin [29] and other classical cadherins [30, 31]. One possible connection between integrins and β-catenin is the integrin-activated, antiapoptotic kinase PKB/Akt. PKB is known to inhibit the activity of glycogen synthase kinase 3-β, a serine kinase that functions directly to reduce β-catenin protein and signaling [32, 33]. It has been established that brain-derived neurotrophic factor (BDNF) binding to TrkB receptors results in a highly specific receptor autophosphorylation [34] and in turn activates the PI3K/Akt pathway [35]. Another target of the PI3K/Akt pathway may be survivin [36]. Survivin is a member of the inhibitor of apoptosis protein (IAP) family, the regulation of survivin by adhesion was examined.

PGE2, downstream of COX-2, can regulate immune function through inhibition of dendritic cell differentiation and T cell proliferation and suppression of the antitumor activity of natural killer cells and macrophages [37].

COX-2 inhibitors blocked tumor growth via an antiangiogenic mechanism [38]. VEGF plays a key role in angiogenesis since it stimulates almost every step in the angiogenic process [39, 40]. Other factors can stimulate angiogenesis include EGF, bFGF, hepatocyte growth factor, interleukin-8, and placental growth factor [41, 42]. Immunohistochemical analysis for VEGF revealed that COX-2 inhibitor prominently suppressed the expression of VEGF in lung metastatic lesion compared with control treatment. In addition, it also reduced VEGF, EGF and bFGF mRNA and protein expression [43]. In vivo, high doses of meloxicam suppressed LM-8 tumor growth and lung metastasis [6].

## Conclusion

In this review, we have tried to encompass the role of COX-2 in the regulation metastasis in osteosarcoma. COX-2 expression and the abundance of its enzymatic product PGE2 play key roles in the development of cancer. Genetic studies support a cause-effect connection between COX-2 and tumorigenesis. COX-2 expression had a poor prognosis with regard to metastasis, and patients with increased COX-2 expression in lung metastases died of the disease. COX-2 expression has also been established as a marker in human osteosarcoma, and COX-2 inhibition has been suggested as a possible way of improving therapeutic outcome [8]. Therefore, COX-2 inhibitors may prove to have a therapeutic role in counteracting the metastasis of osteosarcoma.

## References

[1] Eberhart CE, Coffey RJ, Radhika A, Giardiello FM, Ferrenbach S, DuBois RN (1994). Up-regulation of cyclooxygenase 2 gene expression in human colorectal adenomas and adenocarcinomas. Gastroenterology.

[2] Koga H, Sakisaka S, Ohishi M, Kawaguchi T, Taniguchi E, Sasatomi K (1999). Expression of cyclooxygenase-2 in human hepatocellular carcinoma: relevance to tumor dedifferentiation. Hepatology.

[3] Tucker ON, Dannenberg AJ, Yang EK, Zhang F, Teng L, Daly JM (1999). Cyclooxygenase-2 expression is up-regulated in human pancreatic cancer. Cancer Res.

[4] Hwang D, Scollard D, Byrne J, Levine E (1998). Expression of cyclooxygenase-1 and cyclooxygenase-2 in human breast cancer. J Natl Cancer Inst.

[5] Hida T, Yatabe Y, Achiwa H, Muramatsu H, Kozaki K, Nakamura S (1998). Increased expression of cyclooxygenase 2 occurs frequently in human lung cancers, specifically in adenocarcinomas. Cancer Res.

[6] Naruse T, Nishida Y, Hosono K, Ishiguro N (2006). Meloxicam inhibits osteosarcoma growth, invasiveness and metastasis by COX-2-dependent and independent routes. Carcinogenesis.

[7] Tsujii M, Kawano S, DuBois RN (1997). Cyclooxygenase-2 expression in human colon cancer cells increases metastatic potential. Proc Natl Acad Sci U S A.

[8] Urakawa H, Nishida Y, Naruse T, Nakashima H, Ishiguro N (2009). Cyclooxygenase-2 overexpression predicts poor survival in patients with high-grade extremity osteosarcoma: a pilot study. Clin Orthop Relat Res.

[9] Rodriguez NI, Hoots WK, Koshkina NV, Morales-Arias JA, Arndt CA, Inwards CY (2008). COX-2 expression correlates with survival in patients with osteosarcoma lung metastases. J Pediatr Hematol Oncol.

[10] Tsujii M, DuBois RN (1995). Alterations in cellular adhesions and apoptosis in epithelial cells overexpressing prostaglandin endoperoxidase synthase 2. Cell.

[11] Nzeako UC, Guicciardi ME, Yoon JH, Bronk SF, Gores GJ (2002). COX-2 inhibits Fas-mediate apoptosis in cholangiocarcinoma cells. Hepatology.

[12] Dickens DS, Kozielski R, Leavey PJ, Timmons C, Cripe TP (2003). Cyclooxygenase-2 expression does not correlate with outcome in osteosarcoma or rhabdomyosarcoma. J Pediatr Hematol Oncol.

[13] Masi L, Recenti R, Silvestri S, Pinzani P, Pepi M, Paglierani M (2007). Expression of cyclooxygenase-2 in osteosarcoma of bone. Appl Immunohistochem Mol Morphol.

[14] Chandrasekharan NV, Dai H, Roos KL, Evanson NK, Tomsik J, Elton TS (2002). COX-3, a cyclooxygenase-1 variant inhibited by acetaminophen and other analgesic/antipyretic drugs: cloning, structure, and expression. Proc Natl Acad Sci.

[15] Tsujii M, Kawano S, Tsuji S, Saeaoka H, Hori M, DuBois RN (1998). Cyclooxygenase regulates angiogenesis induced by colon cancer cells. Cell.

[16] PosthumaDeBoer J, Witlox MA, Kaspers GJ, van Royen BJ (2011). Molecular alterations as target for therapy in metastatic osteosarcoma: a review of literature. Clin Exp Metastasis.

[17] Krishnan K, Khanna C, Helman LJ (2005). The biology of metastases in pediatric sarcomas. Cancer J.

[18] Hanahan D, Weinberg RA (2000). The hallmarks of cancer. Cell.

[19] Kashima T, Nakamura K, Kawaguchi J, Takanashi M, Ishida T, Aburatani H (2003). Overexpression of cadherins suppresses pulmonary metastasis of osteosarcoma in vivo. Int J Cancer.

[20] Steeg PS (2006). Tumor metastasis: mechanistic insights and clinical challenges. Nat Med.

[21] Worth LL, Lafleur EA, Jia SF, Kleinerman ES (2002). Fas expression inversely correlates with metastatic potential in osteosarcoma cells. Oncol Rep.

[22] Levings PP, McGarry SV, Currie TP, Nickerson DM, McClellan S, Ghivizzani SC (2009). Expression of an exogenous human Oct-4 promoter identifies tumor-initiating cells in osteosarcoma. Cancer Res.

[23] Saini V, Hose CD, Monks A, Nagashima K, Han B, Newton DL (2012). Identification of CBX3 and ABCA5 as putative biomarkers for tumor stem cells in osteosarcoma. PLoS One.

[24] Pang LY, Gatenby EL, Kamida A, Whitelaw BA, Hupp TR, Argyle DJ (2014). Global Gene expression analysis of canine osteosarcoma stem cells reveals a novel role for COX-2 in tumour initiation. PLoS One.

[25] Klein G, Vellenga E, Fraaije MW, Kamps WA, de Bont ESJM (2004). The possible role of matrix metalloproteinase (MMP)-2 and MMP-9 in cancer, e.g. acute leukemia. Crit Rev Oncol Hematol.

[26] Lee EJ, Choi EM, Kim SR, Park JH, Kim H, Ha KS (2007). Cyclooxygenase-2 promotes cell proliferation, migration and invasion in U2OS human osteosarcoma cells. Exp Mol Med.

[27] Liu B, Shi ZL, Feng J, Tao HM (2008). Celecoxib, a cyclooxygenase-2 inhibitor, induces apoptosis in human osteosarcoma cell line MG-63 via down-regulation of PI3K/Akt. Cell Biol Int.

[28] Ruoslahti E, Reed JC (1994). Anchorage dependence, integrins, and apoptosis. Cell.

[29] Bergin E, Levine JS, Koh JS, Lieberthal W (2000). Mouse proximal tubular cell-cell adhesion inhibits apoptosis by a cadherin-dependent mechanism. Am J Physiol.

[30] Carmeliet P, Lampugnani MG, Moons L, Breviario F, Compernolle V, Bono F (1999). Targeted deficiency or cytosolic truncation of the VE-cadherin gene in mice impairs VEGF-mediated endothelial survival and angiogenesis. Cell.

[31] Tran NL, Adams DG, Vaillancourt RR, Heimark RL (2002). Signal transduction from N-cadherin increases Bcl-2. Regulation of the phosphatidylinositol 3-kinase/Akt pathway by homophilic adhesion and actin cytoskeletal organization. J Biol Chem.

[32] Cook D, Fry MJ, Hughes K, Sumathipala R, Woodgett JR, Dale TC (1996). Wingless inactivates glycogen synthase kinase-3 via an intracellular signaling pathway which involves a protein kinase C. EMBO J.

[33] Cadigan KM, Nusse R (1997). Wnt signaling: a common theme in animal development. Genes Dev.

[34] Klein R, Nanduri V, Jing SA, Lamballe F, Tapley P, Bryant S (1991). The trkB tyrosine protein kinase is a receptor for brainderived neurotrophic factor and neurotrophin-3. Cell.

[35] Douma S, van Laar T, Zevenhoven J, Meuwissen R, van Garderen E, Peeper DS (2004). Suppression of anoikis and induction of metastasis by the neurotrophic receptor TrkB. Nature.

[36] Asanuma H, Torigoe T, Kamiguchi K, Hirohashi Y, Ohmura T, Hirata K (2005). Survivin expression is regulated by coexpression of human epidermal growth factor receptor 2 and epidermal growth factor receptor via phosphatidylinositol 3-Kinase/AKT signaling pathway in breast cancer cells. Cancer Res.

[37] Yang L, Yamagata N, Yadav R, Brandon S, Courtney RL, Morrow JD (2003). Cancer-associated immunodeficiency and dendritic cell abnormalities mediated by the prostaglandin EP2 receptor. J Clin Invest.

[38] Leahy KM, Ornberg RL, Wang Y, Zweifel BS, Koki AT, Masferrer JL (2002). Cyclooxygenase-2 inhibition by celecoxib reduces proliferation and induces apoptosis in angiogenic endothelial cells in vivo. Cancer Res.

[39] Folkman J (2001). Angiogenesis-dependent diseases. Semin Oncol.

[40] Liekens S, De Clercq E, Neyts J (2001). Angiogenesis: regulators and clinical applications. Biochem Pharmacol.

[41] Bellamy WT, Richter L, Sirjani D, Roxas C, Glinsmann-Gibson B, Frutiger Y (2001). Vascular endothelial cell growth factor is an autocrine promoter of abnormal localized immature myeloid precursors and leukemia progenitor formation in myelodysplastic syndromes. Blood.

[42] Yoshida S, Ono M, Shono T, Izumi H, Ishibashi T, Suzuki H (1997). Involvement of interleukin-8, vascular endothelial growth factor, and basic fibroblast growth factor in tumor necrosis factor alpha-dependent angiogenesis. Mol Cell Biol.

[43] Zhao Q, Wang C, Zhu J, Wang L, Dong S, Zhang G (2011). RNAi-mediated knockdown of cyclooxygenase2 inhibits the growth, invasion and migration of SaOS2 human osteosarcoma cells: a case control study. J Exp Clin Cancer Res.

